# Supplementary material for: Utilization of the posterior iliac line for visualizing posterior column screws in obturator oblique view
Source: PLoS One. 2026 Apr 17;21(4):e0347522. doi: 10.1371/journal.pone.0347522 (PMC13089706; doi:10.1371/journal.pone.0347522)
Supplement: Attachment — Submitted filename: Point-by-point response to the reviewers.docx [file pone.0347522.s002.docx]

**Response to Reviewers’ Comments Point by Point**

1. Please complete all items on the Human Participants Research Checklist that are relevant for your submission, by following this link: <http://journals.plos.org/plosone/s/file?id=dc11/PLOSOne_Human_Subjects_Research_Checklist.docx> (Contact us at plosone@plos.org if you cannot access the document.) There may be overlap between the checklist items and other queries listed below; please address any duplicated queries both in your response email and on the checklist itself. Upload the completed Human Participants Research Checklist as file type “Other” when you re-submit your manuscript. This document is for internal journal use only and will not be published if your article is accepted. The requested information will help us to assess whether your submission complies with PLOS ONE’s policies and adheres to applicable reporting standards. Note that your manuscript may be rejected if you provide incomplete or inadequate responses to the checklist questions and that changing the

‘Section/Category’ of your article does not affect this requirement.

**Response:**

We have completed the Human Participants Research Checklist as requested and uploaded it under the file type “Other” during the resubmission of our manuscript.

2. Please provide additional details regarding participant consent. In the ethics statement in the Methods and online submission information, please ensure that you have specified (1) whether consent was informed and (2) what type you obtained (for instance, written or verbal, and if verbal, how it was documented and witnessed). If your study included minors, state whether you obtained consent from parents or guardians. If the need for consent was waived by the ethics committee, please include this information.

If you are reporting a retrospective study of medical records or archived samples, please ensure that you have discussed whether all data were fully anonymized before you accessed them and/or whether the IRB or ethics committee waived the requirement for informed consent. If patients provided informed written consent to have data from their medical records used in research, please include this information.

**Response:**

We have added the requested details regarding participant consent in accordance with the journal’s requirements. Specifically, we have clarified that consent was informed and specified the type of consent obtained. These revisions have been incorporated into the Ethics Statement in the Methods section (lines 79–86) of the revised manuscript.

3. For studies reporting research involving human participants, PLOS ONE requires authors to confirm that this specific study was reviewed and approved by an institutional review board (ethics committee) before the study began. Please provide the specific name of the ethics committee/IRB that approved your study, or explain why you did not seek approval in this case. Once you have amended this/these statement(s) in the Methods section of the manuscript, please add the same text to the “Ethics Statement” field of the submission form (via “Edit Submission”).

**Response:**

We have provided the name of the ethics committee that approved our study and confirmed that the approval was obtained before the study began. This information has been added to the Ethics Statement in the Methods section of the manuscript, as well as to the “Ethics Statement” field of the submission form. Additionally, we have uploaded both the original ethics approval document and its English translation under the file type “Other” for editorial reference.
