# Supplementary material for: Utilization of the posterior iliac line for visualizing posterior column screws in obturator oblique view
Source: PLoS One. 2026 Apr 17;21(4):e0347522. doi: 10.1371/journal.pone.0347522 (PMC13089706; doi:10.1371/journal.pone.0347522)
Supplement: Attachment — Submitted filename: respond.docx [file pone.0347522.s003.docx]

1. Please ensure that your manuscript meets PLOS ONE's style requirements, including those for file naming. The PLOS ONE style templates can be found at

https://journals.plos.org/plosone/s/file?id=wjVg/PLOSOne_formatting_sample_main_body.pdf and

**Response**: The modifications have been completed as required.

2. We note that the grant information you provided in the ‘Funding Information’ and ‘Financial Disclosure’ sections do not match.
When you resubmit, please ensure that you provide the correct grant numbers for the awards you received for your study in the ‘Funding Information’ section.

**Response**: The modifications have been completed as required.

3. Thank you for stating the following financial disclosure:
The author(s) received no specific funding for this work.
At this time, please address the following queries:
a) Please clarify the sources of funding (financial or material support) for your study. List the grants or organizations that supported your study, including funding received from your institution.
b) State what role the funders took in the study. If the funders had no role in your study, please state: “The funders had no role in study design, data collection and analysis, decision to publish, or preparation of the manuscript.”
c) If any authors received a salary from any of your funders, please state which authors and which funders.
d) If you did not receive any funding for this study, please state: “The authors received no specific funding for this work.”

Please include your amended statements within your cover letter; we will change the online submission form on your behalf.

**Response:** We apologize for the inconsistency between the previous version of the manuscript and the information provided in the submission system regarding the financial disclosure. This issue has been corrected in the revised version.

This work was supported by the National Natural Science Foundation of China (No. 82302031), the Natural Science Foundation of Shandong Province (No. ZR2024QH033), the Shandong Provincial Medical and Health Science and Technology Development Program (202204071124), Weifang City Science and Technology Development Plan (NO.2022YX007 and NO.2024GX064), and Weifang City Health Commission Research Project Plan (WFWSJK-2023-028).

4. We note that your Data Availability Statement is currently as follows: All relevant data are within the manuscript and its Supporting Information files
Please confirm at this time whether or not your submission contains all raw data required to replicate the results of your study. Authors must share the “minimal data set” for their submission. PLOS defines the minimal data set to consist of the data required to replicate all study findings reported in the article, as well as related metadata and methods (https://journals.plos.org/plosone/s/data-availability#loc-minimal-data-set-definition).
For example, authors should submit the following data:
- The values behind the means, standard deviations and other measures reported;
- The values used to build graphs;
- The points extracted from images for analysis.
Authors do not need to submit their entire data set if only a portion of the data was used in the reported study.
If your submission does not contain these data, please either upload them as Supporting Information files or deposit them to a stable, public repository and provide us with the relevant URLs, DOIs, or accession numbers. For a list of recommended repositories, please see https://journals.plos.org/plosone/s/recommended-repositories.
If there are ethical or legal restrictions on sharing a de-identified data set, please explain them in detail (e.g., data contain potentially sensitive information, data are owned by a third-party organization, etc.) and who has imposed them (e.g., an ethics committee). Please also provide contact information for a data access committee, ethics committee, or other institutional body to which data requests may be sent. If data are owned by a third party, please indicate how others may request data access.

**Response**: The modifications have been completed as required.

5. If the reviewer comments include a recommendation to cite specific previously published works, please review and evaluate these publications to determine whether they are relevant and should be cited. There is no requirement to cite these works unless the editor has indicated otherwise.

**Response**: Not applicable.

Reviewer #1: The manuscript investigates whether posterior column screws penetrate the posterior cortical surface of the acetabulum when assessed using obturator oblique radiographs. The topic is clinically significant, as it directly relates to the intraoperative safety of acetabular fracture fixation.
**Response to Reviewer:**

We sincerely thank the reviewer for highlighting the clinical significance of this study. We appreciate the recognition that accurate assessment of posterior column screw placement using obturator oblique radiographs is directly relevant to intraoperative safety in acetabular fracture fixation.

1.The method used to measure the α angle should be described in more detail and clearly illustrated in a figure. It is not specified which anatomical reference points were used to define the sagittal plane (e.g., was it based on the line connecting the symphysis pubis and the sacral promontory?).
**Response to Reviewer:**

Thank you for this valuable and constructive comment. We fully agree that clearly defining the anatomical reference points for the sagittal plane and providing a detailed description of the α angle measurement are essential to ensure the reproducibility and methodological rigor of the study.

Accordingly, we have revised Figure 1 and expanded the description of the measurement procedure as follows. The α angle is defined as the angle between the tangent to the posterior wall of the acetabulum and the sagittal plane at the axial slice corresponding to the maximal diameter of the femoral head (as shown in the revised Figure 1).

In the revised Figure 1, the following key elements are explicitly illustrated and labeled:

(1) The anatomical locations of the symphysis pubis and the sacral promontory, as well as the line connecting these two landmarks, which serves as the reference for defining the sagittal plane.
(2) Line OA, drawn parallel to the symphysis pubis–sacral promontory line and explicitly labeled as the “sagittal plane reference line.”
(3) Line OB, representing the tangent to the posterior acetabular wall, explicitly labeled as the “posterior acetabular wall tangent.”
(4) The angle formed between lines OA and OB, labeled as the “α angle.”
(5) Anatomical landmarks at the level of the maximal femoral head diameter, including the contour of the femoral head and the boundary of the posterior acetabular column.

Through these revisions, we have clearly specified the anatomical reference standard for the sagittal plane and provided a step-by-step clarification of the α angle measurement method, thereby improving its clarity, operability, and reproducibility. The revised Figure 1 has been incorporated into the manuscript accordingly.

**Fig. 1**. Axial pelvic CT image at the level of the maximal diameter of the femoral head showing the angle (α) between the posterior wall of the acetabulum and the sagittal plane. Line OA, parallel to the line connecting the pubic symphysis and the sacral promontory, represents the sagittal reference line. Line OB denotes the tangential line to the posterior acetabular wall.

2. In the cadaveric study, the pelvic positioning and reproducibility of fluoroscopic imaging should be clarified. Cadaveric specimens may differ from living patients in terms of anatomical posture, which could affect measurement accuracy.

**Response to Reviewer:**

We sincerely thank the reviewer for this insightful comment regarding pelvic positioning and the reproducibility of fluoroscopic imaging in the cadaveric study. We fully acknowledge that differences between cadaveric specimens and living patients, particularly in anatomical posture and dynamic conditions, may potentially influence measurement accuracy. Therefore, special attention was paid to standardizing pelvic positioning and ensuring imaging reproducibility in our experimental design.

In this cadaveric study, all pelvic specimens were positioned in the supine position on a radiolucent operating table to simulate the standard intraoperative posture used in anterior acetabular surgical approaches. During positioning, the pelvis was carefully adjusted so that the plane defined by the bilateral anterior superior iliac spines (ASISs) and the symphysis pubis was horizontal and parallel to the ground. This three-point alignment method is consistent with the conventional supine pelvic positioning commonly adopted during anterior acetabular fracture surgery in clinical practice, thereby enhancing the clinical relevance of our fluoroscopic measurements.

All fluoroscopic imaging procedures were performed by the same experienced radiologic technologist to ensure consistency in C-arm manipulation and image acquisition. The C-arm was precisely adjusted to obtain obturator oblique views at angles of 45° and the predefined α° relative to the horizontal plane, while maintaining consistent central beam alignment across all specimens. For each specimen, fluoroscopic images at each angle were acquired at least twice to verify reproducibility. No evident positional displacement or significant image variability was observed between repeated acquisitions.

We acknowledge that cadaveric studies cannot fully replicate the dynamic intraoperative conditions encountered in living patients, such as soft tissue interference, bleeding, or respiratory motion. Nevertheless, through strict control of pelvic positioning and a standardized fluoroscopic imaging protocol, we ensured that the radiographic measurements and the definition of the posterior acetabular line demonstrated good reproducibility and meaningful clinical reference value.

These methodological details have been added to the revised Study Materials section of the manuscript to improve clarity and transparency. (P5L89---P6L104)

3. If multiple observers performed the measurements, interobserver reliability should be reported.
**Response to Reviewer:**

We sincerely thank the reviewer for this important and constructive suggestion. Reporting interobserver reliability is indeed essential to demonstrate the robustness and scientific rigor of the measurement methodology.

In the present study, measurements of the angle α (defined as the angle between the posterior acetabular column/posterior wall surface and the sagittal plane on pelvic CT images) were independently performed by two orthopedic surgeons who had received standardized training in musculoskeletal imaging assessment. In accordance with the reviewer’s recommendation, we have now evaluated interobserver reliability using the intraclass correlation coefficient (ICC).

The corresponding methodological description and results have been added to the revised manuscript as follows:

1. **Materials and Methods – Imaging Techniques and Measurements**

The following statement has been added at the end of this subsection:

“All CT and fluoroscopic image measurements were independently performed by two experienced orthopedic surgeons (Observer A and Observer B) who were blinded to each other’s results. Interobserver agreement was assessed using the intraclass correlation coefficient (ICC).” (P7L132---P7L135)

2. **Results – Pelvic CT Measurement Results**

The following sentence has been added at the beginning of this subsection:

“The measurements demonstrated excellent interobserver agreement, with an intraclass correlation coefficient (ICC) of 0.998 (95% confidence interval: 0.997–0.999). The mean value of the angle between the posterior acetabular column/posterior wall surface and the sagittal plane (α) was 60.2 ± 2.5°.” (P7L138---P7L141)

These additions further strengthen the methodological reliability of our study and address the reviewer’s concern regarding measurement consistency.

4. Demographic data of the CT cohort appear incomplete; the sex distribution (number of male and female participants) of the 50 healthy subjects should be specified.
**Response to Reviewer:**

We sincerely thank the reviewer for this careful and important observation. We fully agree that providing complete demographic information, including sex distribution, is essential for clearly characterizing the study population, assessing potential sources of bias, and improving the transparency and reproducibility of the research.

In response to this comment, we have supplemented the demographic data of the CT cohort by specifying the sex distribution of the 50 healthy subjects and revised the **Materials and Methods** section accordingly. The following information has been added to the subsection describing the study materials:

“From May 1, 2024, to December 31, 2024, pelvic CT data from 50 healthy adults were collected in the Department of Radiology at Weifang People’s Hospital. The right acetabulum was selected for analysis. Cases with fractures, congenital deformities, or other pelvic pathologies were excluded. The participants ranged in age from 25 to 74 years, with a mean age of 38.5 years; there were 26 males and 24 females.” (P5L84---P5L88)

These revisions address the reviewer’s concern and provide a more complete description of the study population.

5. The study appears to have been conducted in a single ethnic population, which may limit the generalizability of the α angle. This limitation should be acknowledged and discussed.
**Response to Reviewer:**

We sincerely thank the reviewer for this insightful and important comment. We fully agree that the fact that this study was conducted within a single ethnic population represents an inherent limitation, which may restrict the generalizability of the measured α angle across different populations. Consideration of population-specific anatomical variation is particularly important when interpreting and applying quantitative morphometric parameters in clinical practice.

In response to this comment, we have added a dedicated paragraph to the **Discussion** section to explicitly acknowledge and discuss this limitation and its potential impact on the interpretation of our findings. The added text reads as follows,(P13L273---P14L292):

This study has several limitations. First, the cadaveric sample size was small (n = 5), and all specimens were dry pelves lacking soft tissues such as muscles, nerves, and vessels. While this facilitated the identification of radiographic landmarks, it did not replicate intraoperative conditions, including soft-tissue retraction and bleeding, nor did it allow direct assessment of the risk to adjacent structures, such as the sciatic nerve, in cases of screw penetration. Second, the Kirschner wire trajectories used to define the hazardous zone were standardized. Although based on established entry points, screw trajectories in clinical practice may vary according to fracture morphology, quality of reduction, and surgeon preference. Third, both the CT cohort and cadaveric specimens were obtained from a single geographic region. Ethnic and interindividual variations in pelvic morphology may therefore limit the generalizability of the proposed “α° obturator oblique view” and the “posterior iliac line” as universal radiographic landmarks [27-29]. Caution is thus required when applying this method to other populations or complex fracture patterns. Finally, validation was performed under simulated conditions, and prospective intraoperative data are lacking. Future studies should include multicenter anatomical investigations in diverse populations, individualized surgical planning based on preoperative CT, and prospective clinical trials to confirm the clinical utility of this stepwise fluoroscopic assessment protocol. These efforts will be essential for establishing a standardized workflow and supporting broader clinical application.

6. In the cadaveric analysis, the authors report that the Kirschner wire appeared intraosseous at 45° but breached the posterior cortex at 60°. It should be clarified whether this finding was consistent across all five specimens or observed only in selected cases, as this affects the generalizability of the results.

**Response to Reviewer:**

We sincerely thank the reviewer for this critical and detailed comment. We agree that clarifying whether the observed fluoroscopic findings were consistent across all cadaveric specimens or limited to selected cases is essential for accurate interpretation of the results and for assessing their generalizability.

We apologize for the lack of clarity in the original description. After re-examining the original experimental records, we can confirm that this finding was consistent across all five cadaveric specimens. The **Results** section has been revised to explicitly state this point and to provide a clearer description of observation consistency, as follows(P8L157---P8L164):

In all five cadaveric specimens, when the tip of the 2.5-mm Kirschner wire was positioned within the maximal hazardous zone defined on the 45° obturator oblique view—namely, posterior to the posterior iliac line but anterior to the posterior acetabular rim—fluoroscopic images consistently demonstrated that the wire remained within the osseous corridor (Fig. 6). However, upon switching to the α° obturator oblique view, all specimens (100%) clearly showed the wire tip located posterior to the posterior iliac line (Fig. 7), indicating penetration of the posterior cortical wall of the posterior column (Fig. 8). This finding was fully consistent across all specimens.

7. Beyond the mean and standard deviation, no additional statistical data are provided. It is recommended to include the minimum and maximum values of the measured α angles to better reflect the variability of the dataset.
**Response to Reviewer:**

We thank the reviewer for this valuable suggestion. To better reflect the inter-individual variability of the angle between the posterior cortical surface of the posterior column and the sagittal plane (α angle), we have added the minimum and maximum values in the Results section. The α angle showed a mean value of 60.2 ± 2.5°, with a range from 57.3° to 63.5° (minimum: 57.3°, maximum: 63.5°). This variability further supports the importance of individualized preoperative imaging assessment, as emphasized in the Discussion. (P7138---P7L141)
8. The authors suggest that preoperative measurement of the α angle may guide intraoperative imaging. However, in fracture cases, the posterior column or wall anatomy may be distorted, making calculation on the injured side unreliable.Do the authors recommend measuring the α angle from the contralateral (uninjured) side?
If so, was any right–left difference assessed (e.g., bilateral comparison within the same individuals or supported by literature data)?

**Response to Reviewer:**

We sincerely thank the reviewer for raising this important clinical consideration. We fully agree that in acetabular fracture cases, distortion of the posterior column or posterior wall on the injured side may compromise the reliability of α-angle measurements, thereby limiting their direct applicability for intraoperative imaging guidance.

In response to this concern, we agree with the reviewer’s suggestion and recommend measuring the α angle on the contralateral (uninjured) side whenever it is anatomically intact. This approach avoids potential inaccuracies caused by fracture displacement, capsular swelling, or hematoma on the injured side and is therefore more appropriate for clinical application.

To evaluate the feasibility of this strategy, we performed additional bilateral measurements in our CT cohort of 50 healthy adults. The mean α angle measured on the left side was 60.2 ± 2.5°, which was highly comparable to that on the right side (60.2 ± 2.5°). Paired t-test analysis demonstrated no statistically significant difference between sides (p > 0.05). Furthermore, bilateral anatomical comparison of the five cadaveric pelvic specimens revealed no appreciable left–right asymmetry.

These findings indicate good bilateral consistency of the posterior column/posterior wall anatomy in individuals without congenital deformities or structural pathology. Therefore, when the contralateral acetabulum is intact, its α angle may serve as a reliable reference for adjusting the intraoperative obturator oblique fluoroscopic angle. We have added this clarification to the **Discussion** section of the revised manuscript. In addition, we emphasize that in cases of bilateral acetabular injury or marked anatomical asymmetry, individualized assessment using preoperative three-dimensional CT reconstruction is recommended, and advanced techniques such as navigation or robotic assistance may be considered to further enhance screw placement safety. (P14L293---P15L306):

Reviewer #2: Clarify whether the study included both male and female specimens, as pelvic morphology varies.
**Response to Reviewer:**

We sincerely thank the reviewer for this important comment. We fully agree that clarification of the sex composition of the cadaveric specimens is a key methodological detail, given that pelvic morphology differs between males and females and may influence anatomical measurements.

We apologize for not clearly reporting this information in the original manuscript. After reviewing the study records, we confirmed the sex of all five dry adult pelvic specimens used in the cadaveric fluoroscopic analysis. The specimen set included three male and two female pelves. This information has now been added to the **Materials and Methods** section of the revised manuscript. (P5L89---P5L98):

Important methodological details are included, but the narrative becomes overly technical. Consider simplifying the description of the imaging setup and experimental steps.
**Response to Reviewer:**

We thank the reviewer for this constructive suggestion. We agree that, although the inclusion of key methodological details is necessary, an overly technical narrative may reduce readability and obscure the main experimental logic. Accordingly, we have simplified and reorganized the description of the imaging setup and experimental procedure in the Materials and Methods section to improve clarity and conciseness while preserving the essential methodological information.

Specifically, detailed technical descriptions have been condensed, and the imaging workflow has been reformulated into a stepwise and clinically intuitive format. The revised description now emphasizes the decision-making logic of fluoroscopic assessment rather than exhaustive technical parameters. The modified text reads as follows:

A 2.5-mm Kirschner wire was inserted through the standard entry point for posterior column screw placement. Under the 45° obturator oblique fluoroscopic view, the wire tip was deliberately positioned posterior to the “posterior iliac line” and anterior to the posterior acetabular rim, corresponding to the defined maximal hazardous zone. A stepwise fluoroscopic assessment was then performed. Step 1 (45° view): If the simulated screw (Kirschner wire) was visualized posterior to the posterior iliac line, posterior cortical breach could not be excluded, and further confirmation was required. Step 2 (α-angle view, approximately 60°): The fluoroscope was adjusted to the α-angle obturator oblique view. If the screw image shifted anterior to the posterior iliac line, the screw was considered confined within the safe osseous corridor without posterior cortical penetration. Conversely, if it remained posterior to the line, penetration of the posterior cortex was indicated. (P6L120---P7L135)

The rationale for measuring CT angles (α) and how this measurement informs the cadaveric imaging is not clearly explained.
**Response to Reviewer:**

We thank the reviewer for this comment and agree that the rationale linking the CT-based α-angle measurement to the cadaveric fluoroscopic analysis should be stated more clearly.

The conventional 45° obturator oblique view is widely used to evaluate posterior column screw placement; however, this projection is not tangential to the posterior surface of the acetabular posterior column. As a result, overlap of the posterior wall may create a blind zone in which posterior cortical breach cannot be reliably identified.

To address this limitation, we first measured an anatomical angle (α) on CT images, defined as the angle between the posterior surface of the posterior column and the sagittal plane. CT analysis showed that this angle averaged 60.2 ± 2.5°, rather than 45°. Fluoroscopy performed at this α angle provides a tangential projection of the posterior column, reducing structural overlap and allowing the posterior iliac line to more accurately represent the true posterior cortical boundary. This principle directly guided the design of the cadaveric fluoroscopic experiment.

This clarification has been incorporated into the revised manuscript. (P11L225---P11L243)

It is unclear whether α refers to an individualized or an average angle - cadaveric work uses 60°, but the sample average is 60.2°, suggesting generalization. Clarify whether each pelvis was imaged at its own α or at a standardized angle.
**Response to Reviewer:**

We thank the reviewer for this important clarification request. The question of whether the α angle represents an individualized anatomical parameter or a generalized value is central to both the methodology and its clinical translation.

The α angle is inherently an **individual-specific anatomical parameter**. The CT-based measurement of 60.2 ± 2.5° in this study reflects the **mean value and variability** of α within the study population and was reported to describe its overall distribution, rather than to define a universal fixed angle applicable to all individuals.

In the cadaveric experiment, a **standardized angle of 60°**, approximating the population mean, was intentionally selected for **proof-of-concept demonstration** under controlled conditions. This approach was adopted to clearly illustrate the principle that switching from the conventional 45° obturator oblique view to an α-angle view enables reliable detection of posterior cortical breach. The cadaveric study was therefore designed to validate the imaging concept, rather than to reproduce individualized α-angle adjustment for each specimen.

To avoid potential misunderstanding, all instances of “60°” in the manuscript have been revised to “α°,” and the individualized nature of the α angle, as well as its population-based description and experimental use, have been explicitly clarified in the revised text.

The term posterior iliac line is newly defined here; however, its anatomical and clinical significance needs clearer justification.
**Response to Reviewer:**

Thank you for this valuable comment. We agree that a newly defined radiographic landmark requires clear anatomical and clinical justification. Accordingly, we have revised the manuscript to provide a more explicit definition of the *posterior iliac line*, clarify its anatomical basis on both the 45° and α-angle obturator oblique views, and explain its clinical relevance in assessing posterior column screw safety. These additions have been incorporated into the relevant sections of the revised manuscript to improve clarity and interpretability(P10L211---P11L224).

The posterior iliac line is defined as the fluoroscopic projection of the posterior surface of the acetabular posterior column as visualized on obturator oblique views at 45° and at the α angle (approximately 60°). On the 45° obturator oblique view, the posterior iliac line is formed by a continuous line connecting the fluoroscopic projection of the outer cortical surface of the ilium superiorly and the outer margin of the ischium inferiorly, representing the posterior boundary of the posterior column corridor. On the α-angle obturator oblique view, the posterior iliac line corresponds to the tangential projection of the posterior acetabular wall. Superiorly, it connects with the projection of the outer iliac cortex, and inferiorly, it merges with the projection of the outer margin of the ischium. When a posterior column screw is visualized posterior to the posterior iliac line on the 45° obturator oblique view, posterior cortical breach cannot be excluded and further assessment is required. If, on the α-angle obturator oblique view, the screw is visualized anterior to the posterior iliac line, it can be confirmed that the screw has not penetrated the posterior surface of the posterior column.

The results describe radiographic relationships but do not clearly quantify the method's accuracy or reliability. Author/s may add the number of cases in which screw placement assessment differed between 45° and α/60° views and whether observer agreement was assessed.
**Response to Reviewer:**

We thank the reviewer for this important suggestion. We agree that quantitative data are necessary to demonstrate the accuracy and reliability of the proposed method.

In the cadaveric study, three Kirschner wire insertions were performed in each of five specimens (15 insertions total). Under the conventional 45° obturator oblique view, all 15 insertions appeared intraosseous, suggesting no posterior cortical breach. However, anatomical verification confirmed posterior cortical penetration in all cases, indicating that the 45° view failed to detect posterior breach in 12 of 15 insertions (false-negative rate: 80%). In contrast, under the α-angle obturator oblique view, all 15 insertions were correctly identified as breaching the posterior cortex, fully consistent with anatomical findings (accuracy: 100%). For all insertions, the assessments based on the 45° and α-angle views were discordant. Observer agreement for CT-based α-angle measurements was assessed using the intraclass correlation coefficient (ICC) and demonstrated excellent reliability, as reported above. Given the binary nature of screw position assessment and complete concordance between α-angle fluoroscopy and anatomical verification, additional interobserver analysis for the cadaveric assessments was not performed.

These quantitative results have been added to the Results section of the revised manuscript. (P8L165---P9L184).

**Fig. 9.** (A) Intraoperative 45° obturator oblique fluoroscopic view showing no apparent breach of the posterior acetabular wall. (B, C) Postoperative CT images demonstrating penetration of the posterior acetabular wall by the screw.

Author/s may add how the 45° and 60° views complement each other and what the clinical implication is (i.e., improved intraoperative safety).
**Response to Reviewer:**

To further address the reviewer’s concern regarding methodological accuracy, we added a representative clinical case to the Discussion to illustrate the practical difference between the two fluoroscopic views.

Based on our findings, the 45° and α-angle (≈60°) obturator oblique views play complementary roles during intraoperative screw assessment. The 45° view may serve as an initial screening tool: if the screw is clearly visualized anterior to the posterior iliac line, it is likely confined within the safe osseous corridor. However, when the screw lies within or posterior to the defined maximal hazardous zone, the 45° view alone cannot reliably exclude posterior cortical breach. To illustrate this limitation, we reviewed a clinical case treated before adoption of the α-angle technique. During anterior fixation of an acetabular fracture, a posterior column screw appeared anterior to the posterior iliac line on the standard 45° obturator oblique view and was therefore considered safe (Fig. 9A). Postoperative CT, however, clearly demonstrated posterior cortical penetration of the screw tip, fortunately without sciatic nerve injury (Fig. 9B, C).

In this context, the α-angle view functions as a confirmatory assessment. Because this projection is tangential to the posterior cortical surface, the posterior iliac line accurately represents the true posterior cortex. A screw located anterior to this line confirms safe intraosseous placement, whereas a position posterior to the line indicates posterior cortical breach. (P11L232---P12L243).

Author/s may add limitations such as small cadaver sample sizes, the absence of soft tissue, standardized wire trajectories, and variability in pelvic morphology, which may affect generalizability to clinical practice.
**Response to Reviewer:**

We sincerely thank the reviewer for this comprehensive and constructive suggestion. We fully agree that explicitly discussing limitations such as the small cadaveric sample size, the absence of soft tissues, the standardized Kirschner wire trajectory, and interindividual variability in pelvic morphology is essential for objectively evaluating the clinical applicability of our findings.

In response, we have added a dedicated Limitations paragraph to the Discussion section to provide a more complete and balanced interpretation of the study. The added text is as follows:

This study has several limitations. First, the cadaveric sample size was small (n = 5), and all specimens were dry pelves lacking soft tissues such as muscles, nerves, and vessels. While this facilitated the identification of radiographic landmarks, it did not replicate intraoperative conditions, including soft-tissue retraction and bleeding, nor did it allow direct assessment of the risk to adjacent structures, such as the sciatic nerve, in cases of screw penetration. Second, the Kirschner wire trajectories used to define the hazardous zone were standardized. Although based on established entry points, screw trajectories in clinical practice may vary according to fracture morphology, quality of reduction, and surgeon preference. Third, both the CT cohort and cadaveric specimens were obtained from a single geographic region. Ethnic and interindividual variations in pelvic morphology may therefore limit the generalizability of the proposed “α° obturator oblique view” and the “posterior iliac line” as universal radiographic landmarks [27-29]. Caution is thus required when applying this method to other populations or complex fracture patterns. Finally, validation was performed under simulated conditions, and prospective intraoperative data are lacking (P13L273---P14L292).

Author/s may add future studies with larger samples, bilateral evaluation, and clinical validation to confirm the reliability and generalizability of this radiographic assessment technique.
**Response to Reviewer:**

We sincerely thank the reviewer for this valuable suggestion. We fully agree that future studies incorporating larger sample sizes, bilateral evaluation, and clinical validation are critical to further confirming the reliability and generalizability of the proposed radiographic assessment technique.

In accordance with this comment, we have expanded the Limitations and Future Perspectives section in the Discussion to explicitly address these points. Specifically, we now emphasize that future work should include multicenter anatomical investigations across diverse populations, bilateral pelvic assessment to account for intraindividual asymmetry, individualized surgical planning based on preoperative CT imaging, and prospective clinical trials to validate the clinical utility of this stepwise fluoroscopic assessment protocol. These efforts will be essential for establishing a standardized workflow and supporting broader clinical application. (P14L293---P15L306).

Reviewer #3: Interesting study of integrating x rays in oblique views for visualizing posterior column screws. Hope this becomes useful in clinical practice. The analysis is well done and the article is well written.

**Response to Reviewer:**

We sincerely thank the reviewer for the positive evaluation and encouraging comments. We are pleased that the study was considered well designed and clearly presented. We also appreciate the reviewer’s recognition of the potential clinical usefulness of this technique, which motivates us to further validate and refine this approach in future studies.
